# Dia2 Controls Transcription by Mediating Assembly of the RSC Complex

**DOI:** 10.1371/journal.pone.0021172

**Published:** 2011-06-20

**Authors:** Edward J. Andress, Roman Holic, Mariola J. Edelmann, Benedikt M. Kessler, Veronica P. C. C. Yu

**Affiliations:** 1 Eukaryotic Chromatin Dynamics Group, MRC Clinical Sciences Centre, Imperial College Hammersmith Campus, London, United Kingdom; 2 Ubiquitin Proteolysis Group, Central Proteomics Facility, Nuffield Department of Clinical Medicine, Centre for Cellular and Molecular Physiology, Oxford University, Oxford, United Kingdom; 3 Department of Medical and Molecular Genetics, King's College London School of Medicine, Guy's Hospital, London, United Kingdom; University of Hong Kong, Hong Kong

## Abstract

**Background:**

Dia2 is an F-box protein found in the budding yeast, *S. cerevisiae.* Together with Skp1 and Cul1, Dia2 forms the substrate-determining part of an E3 ubiquitin ligase complex, otherwise known as the SCF. Dia2 has previously been implicated in the control of replication and genome stability via its interaction with the replisome progression complex.

**Principal Findings:**

We identified components of the RSC chromatin remodelling complex as genetic interactors with Dia2, suggesting an additional role for Dia2 in the regulation of transcription. We show that Dia2 is involved in controlling assembly of the RSC complex. RSC belongs to a group of ATP-dependent nucleosome-remodelling complexes that controls the repositioning of nucleosomes. The RSC complex is expressed abundantly and its 17 subunits are recruited to chromatin in response to both transcription activation and repression. In the absence of Dia2, RSC-mediated transcription regulation was impaired, with concomitant abnormalities in nucleosome positioning.

**Conclusions:**

Our findings imply that Dia2 is required for the correct assembly and function of the RSC complex. Dia2, by controlling the RSC chromatin remodeller, fine-tunes transcription by controlling nucleosome positioning during transcriptional activation and repression.

## Introduction

Nucleosomes pose formidable barriers to transcription. Sophisticated chromatin remodellers have evolved to specifically coordinate and fine-tune the accessibility of DNA to the basic transcription machinery. The RSC complex is the most abundant of such chromatin remodellers in eukaryotes. Comprised of 17 subunits, the RSC complex is an ATP-dependent chromatin remodelling complex that has been demonstrated to slide or disassemble nucleosomes [1], [2]. In addition to transcription, the RSC complex is also involved in many other aspects of chromatin metabolism including DNA replication and repair [3]–[5]. In the budding yeast *Saccharyomyces cerevisiae*, the RSC complex has been shown to bind to transcribed parts of the genome and control nucleosome distribution in response to transcription [6], [7]. Not all RSC subunits are essential for survival. In yeast, the RSC complex can exist as sub-modules without the presence of all 17 subunits [8]. However, what controls assembly of the RSC complex *in vivo* is currently not clear.

Dia2 is part of an E3 ubiquitin ligase (otherwise known as SCF^Dia2^) that has been proposed to constitute part of the replisome progression complex [9], [10]. Dia2 has been demonstrated to be essential for the maintenance of genome integrity during S phase [11]–[13]. Though the exact mechanism remains unclear, it is believed that Dia2 acts at stalled forks. Interestingly, the S phase damage checkpoint protects Dia2 from degradation [14], [15].

In investigating the relationship between Dia2 and chromatin, we describe here a novel aspect of Dia2 function in transcription. This correlation is dependent on Dia2's ability to modify assembly of the RSC complex, hence the ability to influence nucleosome distribution during transcription.

## **Results**

### Dia2 interacts genetically with the RSC complex

**Figure 1:**
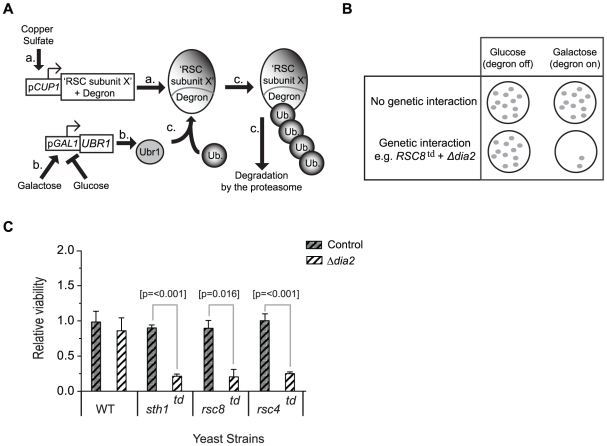
Dia2 genetically interacts with the RSC complex. 1A) Experimental design Each RSC degron strain harbours two inducible genes: a) One is a subunit of the RSC complex, fused to the N-end rule degron motif and driven by the *CUP1* promoter. Addition of copper sulfate to the media induces expression of the degron construct (denoted by the ^td^ suffix). b) The second is the N-end rule ubiquitin ligase, Ubr1, driven by the *GAL1* promoter. Addition of galactose causes Ubr1 expression and consequent ubiquitylation of the degron-tagged RSC subunit and causes its proteasome-dependent degradation. c) Degradation by this mechanism is optimal at 37°C and occurs at a reduced level at 30°C. Complete degradation of the RSC-degron subunits is not desired, as most RSC subunits are essential for cell survival. 1B) Genetic screen design Yeast strains harbouring degron-tagged RSC subunits were transformed with a *dia2* knock-out cassette and grown in either dextrose (degron-repressing) or galactose (degron-activating) media. In parallel, a control set of transformations was conducted in the same way, using an irrelevant knock-out cassette (Δ*suc2* control). If there was synthetic lethality between the RSC degron mutant and Δ*dia2*, the number of viable colonies will be reduced in galactose but not in dextrose. 1C) Numerical analysis of the experiment described in 1B Synthetic lethality of Δ*dia2* was observed with the *sth1^td^*, *rsc8^td^* and *rsc4^td^* mutants. The interaction is specific as viability was only reduced when Δ*dia2* was transformed under degron-activating (‘Gal’/YPG) growth conditions. To normalise for the effects of carbon source on transformation efficiency, all colony counts were normalised against the value for the corresponding dextrose samples (relative viability =  no. of colonies on YPG/ no. of colonies on YPD). Data presented represents three sets of technical replicates (n = 3).

Dia2 interacts genetically with multiple chromatin modifiers [16], [17]. Blake *et al.* in particular, demonstrated that Δ*dia2* was synthetically lethal with deletion in genes involved in maintaining chromatin structure such as Htz1, Hst4, Swr1, Sgf29 and Npt1. This opens the possibility that in addition to its role in replication [12], [13], [18], Dia2 may be involved in other aspects of chromatin metabolism such as the control of transcription. Whilst investigating proteins, which exhibit synthetic lethality with RSC complex, we noticed a strong genetic interaction between several subunits of the RSC complex and Dia2 (Fig. 1). Using an assay that made use of inducible degron mutants of RSC complex components [11] (see Figs 1A and 1B for experimental design), we found that deletion of *dia2* was synthetically lethal with *sth1^td^*, *rsc8^td^* and *rsc4 ^td^* subunits of the RSC complex upon induction of the Ubr1 ubiquitin ligase that targets the degron fusion proteins for destruction, at the semi-permissive temperature of 30 degrees (Fig. 1). Results of all other RSC subunits tested are shown in supplementary Fig. S1.

### Dia2 promotes RSC-mediated transcriptional regulation

**Figure 2:**
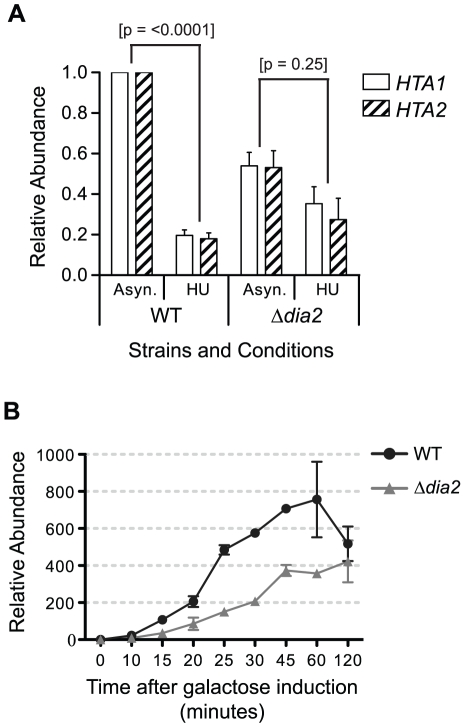
Dia2 is required for regulation of transcription. 2A) Dia is required of basal expression and repression of histone H2A/H2B genes Total RNA was extracted from yeast cells grown in YPD either in the absence (asyn) or after addition of 200 mM of hydroxyurea (HU) for 40 minutes to induce repression. RNA was reverse transcribed and analysed by quantitative PCR. Samples were normalised against expression of the *ACT1* gene. 2B) Efficient induction of *GAL1* expression requires Dia2 Yeast cells grown exponentially in raffinose were induced for *GAL1* expression by the addition of galactose. Samples were taken at various time intervals for RNA extraction followed by qPCR. Signals from triplicate samples were amplified using *GAL1* primers and normalised against those obtained using *ACT1* primers. Wild-type (WT) cells showed a rapid increase in *GAL1* signal from 15 minutes. This peaked at around 60 minutes. In Δ*dia2* cells, *GAL1* expression did not increase until 20 minutes, and the observed maximal level of induction was about half of that achieved in wild-type cells.

The RSC complex is involved in both transcription activation and repression [2], [6]. We first examined transcriptional control at the histone gene locus *HTA1/HTB1* where the RSC complex has been shown to participate in repression of histone gene transcription [6]. Histone gene expression is restricted to S phase and is repressed by hydroxyurea, which inhibits DNA replication [19]. We found that *HTA1* expression in asynchronously growing cells was reduced in the *dia2* deletion mutant, Δ*dia2* (Fig. 2A). In wild-type cells, hydroxyurea suppresses histone gene expression to 20% of the level observed during asynchronous growth. While at a somewhat lower levels to start with, reduction in response to HU was hardly observed. This suggests that Dia2 is required for both, full transcription of *HTA1/HTB1* in undisturbed conditions, as well as their transcriptional repression in response to HU. Because the RSC complex has not yet been shown to act in histone gene induction, and the inability to repress *HTA1* in response to HU might be masked by the defect in gene induction, we examined histone H3/H4 genes transcription. Histone H3 and H4 genes are expressed from two loci, *HHT1/HHF1* and *HHT2/HHF2* [20]. Both these loci have bidirectional promoters and are regulated in a similar fashion to the *HTA1/HTB1* locus in a cell cycle dependent manner [21]. Repression of histone H3 and H4 is RSC-dependent [6]. While *HHT1/HHF1* and *HHT2/HHF2* expression in exponentially growing cells was undisturbed in the Δ*dia2* mutant, repression in response to HU was no longer observed (Fig. S2). Thus, Dia2 is required for regulation of the genes encoding all four core histones.

The Δ*dia2* mutant shows a delay in transiting through the S/G2 phases of the cell cycle due to accumulation of DNA damage [15]. This could impact indirectly on histone gene transcription. However, Rad53 phosphorylation is still induced by HU in the Δ*dia2* mutant [17]. Therefore, the inability of Δ*dia2* to suppress histone transcription in response to HU exposure suggests a checkpoint-independent role of Dia2 in transcriptional repression. To confirm a role of Dia2 in transcription, we examined the *GAL1/GAL10* locus that is regulated independently of cell cycle progression and the DNA damage response.

The RSC complex has previously been demonstrated to be directly recruited to the bidirectional *GAL1/GAL10* promoter and downstream open reading frames (ORFs) during gene induction [7], [22], [23]. In wild-type cells, the addition of galactose to cells growing in raffinose rapidly induced *GAL1* transcription (Fig. 2B). This response was delayed and yielded only reduced *GAL1* induction in the Δ*dia2* mutant, suggesting a role in transcriptional activation. Dia2 is therefore required for both effective transcriptional activation and repression of genes, which are known to require the RSC complex for regulation.

### Dia2 is recruited to RSC-regulated promoter regions

**Figure 3:**
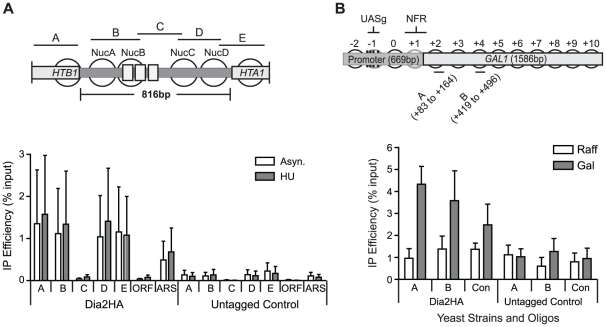
Dia2 is bound to chromatin during transcription. 3A) Dia2 binds to the *HTA1/HTB1* promoter. Chromatin immunoprecipitation was performed using haemagglutinin epitope-tagged Dia2 (Dia2-HA) and untagged control strains. Precipitated DNA was analysed by quantitative PCR using primers spanning the *HTA1/HTB1* promoter (A to E) as depicted (top). qPCR analysis of Dia2 recruitment to the *HTA1/HTB1* locus in the absence (Asyn.) or presence of hydroxyurea (HU) are plotted (bottom). Each data point is normalised to its corresponding input. Signals obtained from an untagged control sample are plotted on the right for comparison. ‘ORF’ denotes a primer located within the *HTA1* open reading frame. ARS denotes primers amplifying an origin of replication (*ARS428*) close to *HTA1/HTB1*. 3B) Dia2 is recruited to the *GAL1* promoter and ORF during gene induction Chromatin immunoprecipitation of HA-tagged Dia2 was carried out in wild-type cells grown either in raffinose or 20 minutes after galactose induction. Positions of primers (A and B) for *GAL1* used to amplify the precipitated DNA are depicted in the top panel. Dia2 binds to *GAL1* only during gene induction (in galactose but not in raffinose). In the presence of raffinose, the level of Dia2 recruitment is insignificant (comparable to background levels in the untagged control). Each data point is normalised to its corresponding input. Signal obtained from an untagged control strain are depicted on the right for comparison. ‘Con’ denotes a transcriptionally silent subtelomeric region on chromosome II.

To investigate whether Dia2 is directly involved in transcriptional regulation of RSC target genes, we carried out chromatin immunoprecipitation experiments. Genomically epitope-tagged Dia2 was recruited to the *HTA1/HTB1* promoter. Recruitment occurred at equal levels in untreated and HU treated cells (Fig. 3A), consistent with both the reduced levels of expression in undisturbed conditions as well as defective repression we observed earlier in Δ*dia2* cells. These findings are consistent with the possibility that Dia2 is constitutively associated with the *HTA1/HTB1* promoter.

We next tested Dia2 binding to the *GAL1/GAL10* locus. Dia2 was specifically recruited to the *GAL1* promoter during gene induction after galactose addition (Fig. 3B). We did not detect Dia2 at the *GAL1* locus in cells grown in raffinose when transcription is not induced. This suggests that, at least at the *GAL1* promoter, Dia2 is not a constitutive component. Rather, Dia2 is recruited to the promoter as part of the transcriptional induction process. Together, the results of our chromatin immunoprecipitation analysis demonstrate that Dia2 is present at promoters of genes whose regulation depends on Dia2.

### Dia2 controls RSC complex assembly

**Figure 4:**
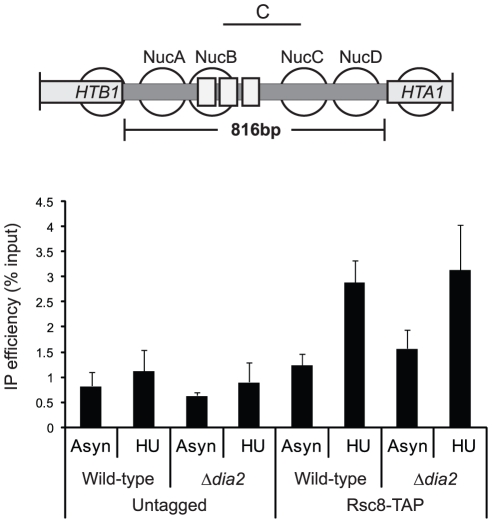
Rsc8 was efficiently recruited to the *HTA1/HTB1* locus in the Δ*dia2* mutant. Chromatin immunoprecipitation of tandem affinity purification (TAP)-tagged Rsc8 was carried out in the wild-type and the Δ*dia2* mutant and compared to results from an untagged control. Precipitated DNA was analysed by quantitative PCR using primer pair C spanning the *HTA1/HTB1* promoter as depicted in Fig. 3A. Samples grown logarithmically in the absence (Asyn) or presence of hydroxyurea (HU) are compared. Each data point is normalised to its corresponding input.

Histone gene repression involves recruitment of the HIR nucleosome assembly complex, which in turn directs association of the RSC complex [22], [23]. To investigate the mechanism by which Dia2 contributes to regulation of the locus, we examined recruitment of the Rsc8 subunit of the RSC complex to the *HTA1/HTB1* locus, which has previously been characterized as downstream event of HIR recruitment [6]. Chromatin immunoprecipitation analysis revealed that Rsc8 association with the *HTA1/HTB1* promoter was unaffected in the Δ*dia2* mutant (Fig. 4). This suggests that the upstream HIR complex pathway was intact and recruitment at least of the Rsc8 subunit of the RSC complex to chromatin did not require Dia2.

**Figure 5:**
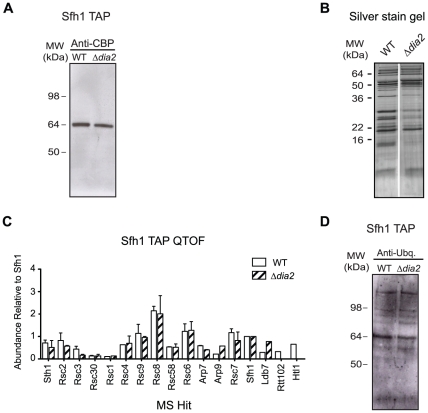
Dia2 is required for proper assembly of the RSC complex. Tandem affinity purification (TAP) and subsequent analysis by tandem mass spectrometry was performed using an Sfh1-TAP expression strain in both the wild-type and Δ*dia2* backgrounds. 5A) Sfh1 pull-down of the RSC complex Western blotting shows efficient immunoprecipitation of TAP-tagged Sfh1 complexes from both the wild-type and Δ*dia2* strains. Sfh1 (recognised by the anti-calmodulin-binding protein antibody, anti-CBP) migrates at 64 kDa, as expected. 5B) RSC complex purified from the Δ*dia2* differed from the wild-type Silver stained gel showing proteins purified following TAP purification in the wild-type and Δ*dia2* strains (Δ*dia2* showed quantitative differences in immunoprecipitated proteins compared to wildtype). 5C) RSC complex in the Δ*dia2* mutant lacked subunits Semi-quantitative plot of differential abundance of protein complex components obtained by evaluating their exponentially modified protein abundance index (EmPAI) following analysis with tandem mass spectrometry. Error bars represent standard error of the mean from two biological replicates. 5D) Pattern of ubiquitylation did not differ between wild-type and Δ*dia2* RSC complexes Western blotting of Sfh1 immunopurified RSC complexes using an anti-ubiquitin antibody. Multiple bands potentially representing ubiquitylated subunits of the RSC complex are present equally in wild-type and Δ*dia2* samples.

Because Rsc8 recruitment to chromatin was intact in the Δ*dia2* mutant, to further investigate the relationship between Dia2 and the RSC complex, we analysed RSC complex assembly in the Δ*dia2* strain. We performed tandem-affinity purification of the RSC complex using the TAP-tagged Sfh1 subunit as bait. In the wild-type strain, all members of the RSC complex were co-purified (Fig. 5A, B). We applied a comparative proteomics approach using nano-UPLC-MS/MS to identify the RSC subunits and to gain a semi-quantitative readout of the protein complex composition. We found that in the Δ*dia2* mutant, several members of the RSC complex were less abundant or missing (Fig. 5B). These included Htl1, Rtt102 and Rsc3 (Fig. 5C, Table 1). This was not because of inefficient Sfh1 immunoprecipitation in the Δ*dia2* mutant (Fig. 5A, Fig. S4). These results suggest that Dia2 is required for assembly of the full RSC complex, containing all of its known subunits.

**Table 1:** Summary of LC MS/MS data from peptides obtained from immunoprecipitation of RSC complexes from wild-type and Δ*dia2* strains.

	WT				Δ*dia2*			
	EmPAI	Unique peptides	Coverage (%)	Mowse score	EmPAI	Unique peptides	Coverage (%)	Mowse score
Sth1	0.32	71	49	299	0.37	74	55	289
Rsc1	0.06	21	23	53	0.06	18	22	56
Rsc2	0.27	38	43	519	0.27	37	49	301
Rsc3	0.31	35	33	182	0.06	24	22	47
Rsc30	0.06	23	27	52	0.03	21	20	38
Rsc4	0.35	21	39	464	0.46	19	33	463
Rsc9	0.85	30	61	692	0.45	24	48	365
Rsc8	1.29	34	53	877	1.27	33	62	944
Rsc58	0.3	29	58	226	0.3	22	49	218
Rsc6	0.86	31	61	469	0.75	29	51	380
Arp7	0.33	21	59	240	0.19	23	49	139
Arp9	0.12	15	34	53	0.26	17	43	61
Rsc7	0.74	26	60	374	0.54	23	55	354
Sfh1	0.55	12	50	410	0.45	24	62	257
Ldb7	0.16	10	64	49	0.35	4	38	108
Rtt102	0.18	9	70	168	-	-	-	-
Htl1	0.36	7	61	34	-	-	-	-

Given that Dia2 is part of an E3 ubiquitin ligase complex, we postulated that its effect on the RSC complex might be dependent on its ubiquitylating properties. Our mass spectrometric data contained evidence that subunits from the RSC complex were ubiquitylated (data not shown). We surmised that if Dia2-mediated ubiquitylation of RSC subunits had a direct impact on RSC complex assembly, the RSC ubiquitylation pattern would be altered in the Δ*dia2* mutant. To test this, we immunoprecipitated the RSC complex from wild-type or the Δ*dia2* mutant strain using TAP-tagged Sfh1 and performed western blotting using an anti-ubiquitin antibody (Fig. 5D). Sfh1 was adequately expressed and immunoprecipitated in both wild-type and Δ*dia2* samples. No obvious difference in the pattern of ubiquitylation was observed between wild-type and the Δ*dia2* sample. This suggests that ubiquitin ligases in addition to Dia2 are targeting the RSC complex. Dia2 might also promote ubiquitylation of RSC complex components, but the ubiquitylation events may have been masked by those by other ubiquitin ligases in our Western blot analysis. Alternatively, we cannot exclude that RSC complex assembly is controlled by Dia2 via ubiquitylation of proteins different from RSC subunits, or in a manner independent of SCF^Dia2^ ubiquitin ligase activity.

**Figure 6:**
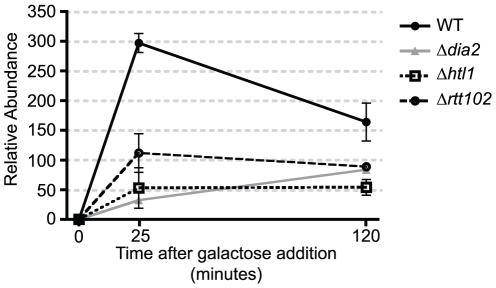
Δ*htl1* and Δ*rtt102* mutants were defective in *GAL1* transcription. Yeast cells grown exponentially in raffinose were induced for *GAL1* expression by the addition of galactose. Samples were taken at various time intervals for RNA extraction followed by qPCR. Signals from triplicate samples were amplified using *GAL1* primers and normalised against those obtained using *ACT1* primers. Unlike wild-type cells, Δ*htl1* and Δ*rtt102* cells were defective in *GAL1* expression similar to Δ*dia2* cells. This was most evident at 25 minutes. The observed steady state at 120 minutes was about half of that achieved in wild-type cells in all of the mutants.

If transcriptional defects in the absence of Dia2 are indeed due to partial RSC complex assembly, then equivalent RSC complex mutants should display similar defects in gene regulation. The RSC complex in the Δ*dia2* strain lacked the two non-essential Htl1 and Rtt102 subunits (Rsc3 was an essential subunit). We therefore examined transcription of the *GAL1* gene in Δ*htl1* and Δ*rtt102* mutants (Fig. 6). Both Δ*htl1* and Δ*rtt102* were impaired in their ability to induce *GAL1* at 25 minutes. The magnitude of the defect was reminiscent of the Δ*dia2* mutant defect, which is consistent with the possibility that Dia2 acts in transcriptional regulation by promoting RSC complex assembly.

### Dia2 is required for correct nucleosomal patterning

**Figure 7:**
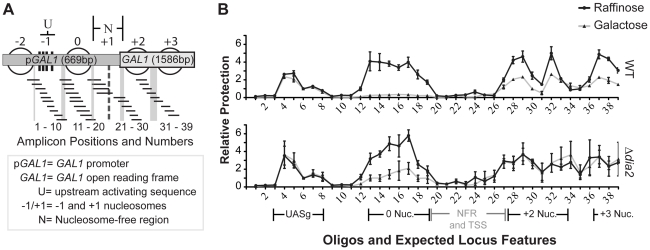
Dia2 is required for efficient eviction and positioning of nucleosomes at *GAL1.* Micrococcal nuclease protection assays were performed using wild-type (WT) and Δ*dia2* strains. Cells were grown in raffinose-containing medium (circles) and GAL1 expression was induced for 20 minutes by galactose addition (triangles). Mono-nucleosomal sized DNA-fragments were obtained by micrococcal nuclease digest. DNA was then extracted before analysis by qPCR (Fig. 7B) using the panel of primers along the *GAL1* locus as shown in Fig. 7A. The virtually complete removal of the promoter nucleosome (0 Nuc) following gene induction in wild type cells was considerably reduced in the Δ*dia2* strain. Reduced nucleosome occupancy was also evident at positions corresponding to the +2 and +3 nucleosome after galactose addition in the presence but not in the absence of Dia2. Error bars represent standard error of the mean of three biological replicates. Galactose samples were compared by a 2-way ANOVA to give statistical significance to eviction defects at the 0, +2 and +3 nucleosomes. WT vs Δ*dia2, p*<0.0001, <0.0001, 0.003, respectively.

If the abnormal composition of the RSC complex in the absence of Dia2 had functional significance, given the known function of the RSC complex as a chromatin remodeller, the observed transcriptional defects in the Δ*dia2* mutant could be due to a disturbance in nucleosome positioning. To address this, we mapped nucleosome positions using an assay based on protection from micrococcal nuclease by nucleosomes [24], [25] at the bi-directional *GAL1/GAL10* promoter (Fig. 7A). When cells were induced with galactose, the *GAL1* upstream promoter nucleosome (marked Nuc 0, Fig. 7B) was evicted in the wild-type strain as expected [25]. Eviction of this nucleosome was markedly reduced in the Δ*dia2* mutant. This was in keeping with, and could explain, the observed transcriptional defect (Fig. 2B). Interestingly, nucleosome boundaries were less sharply defined in the Δ*dia2* mutant along the *GAL1* open reading frame (+2 and +3 nucleosomes) under both uninduced (raffinose) and induced (galactose) conditions. Furthermore, the reduction in nucleosome occupancy at these positions observed in wild type cells in response to *GAL1* induction was no longer detectable in the absence of Dia2. It has recently been reported [7] that RSC mutants showed alterations to a nuclease hypersensitive area (HS) flanking the UASg site (Fig. 7A). In our analysis, we did not observe changes to the nucleosome occupancy around UASg in the Δ*dia2* mutant. This could be due to the fact that a RSC core complex, comprising its essential subunits, was assembled independently of Dia2, so that the Δ*dia2* mutant may cause a distinct defect due to the missing RSC sub-module.

We also observed nucleosome positioning abnormalities at the *HTA1/HTB1* locus, including less sharply defined nucleosome positions and reduced changes in response to HU treatment (Fig. S3). This could contribute to the reduced transcription at this locus in the Δ*dia2* strain during asynchronous growth and its defective repression (Fig 2A). Together our results show that the correct nucleosome patterning and nucleosome repositioning during transcriptional regulation are under the influence of Dia2, likely because of its requirement for correct assembly of the RSC chromatin remodelling complex.

## Discussion

The F-box protein Dia2 has been associated with replication stress response and is considered part of the replisome progression complex responsible for fork stability especially during traversing through regions of the genome prone to fork stalling and DNA damage. Recently, Dia2 has also been linked to part of the intra-S phase DNA damage checkpoint [10], [13], [14], [26].

In this study, we demonstrated that Dia2 genetically interacts with the RSC complex in the context of transcription and is required for complete RSC complex assembly. RSC is an essential chromatin remodelling complex involved in multiple aspects of chromatin metabolism during transcription, recombination, repair, and replication. We showed that Dia2 interacted genetically with subunits of the RSC complex and proposed a model whereby SCF^Dia2^ recruitment to specific regions of chromatin during transcriptional events modulated transcription by controlling nucleosome dynamics. We demonstrated that Dia2 was recruited to transcription units and was required for efficient transcription, transcriptional induction and transcriptional repression at the respective loci. We initially examined HU-mediated repression at histone gene loci. We found that Dia2 was required for repression of histone genes (Fig. 2A and Fig. S2). Because of the known role of RSC in histone gene repression we suggest that the failure of repression in the Δ*dia2* mutant was due to the defects in RSC complex that we have identified. In addition, basal expression levels at the *HTA1/HTB1* locus, but not at the *HHT1/HHF1* and *HHT2/HHF2* loci, depended on Dia2. We do not currently know the reason for this difference. It could be due to a RSC requirement for active *HTA1/HTB1* transcription that is not shared at the *HHT1/HHF1* and *HHT2/HHF2* loci where another chromatin remodelling complex might be able to take on a role in activation of gene expression. Alternatively, there might be an unknown Dia2-dependent substrate that is required to transcribe the H2AH2B but not the H3H4 genes (hence the induction defect was only seen at *HTA1* and *HTA2*). Further experiments are required to investigate these possibilities.

Given the requirement for Dia2 in maintaining replicative fidelity and that the Rad53 checkpoint is active in Δ*dia2* cells [17], it is difficult to entirely disentangle transcriptional effects from the known replicative defects. We therefore also examined the *GAL1/GAL10* locus, which is known to come under control of the RSC complex, but whose regulation is cell cycle independent. We observed a delay and an overall inefficiency during gene induction in Δ*dia2* cells. The RSC complex is known to bind the *GAL1/GAL10* promoter [7], [22]. Using a temperature-sensitive *RSC* mutant, *rsc3-1ts*, it has been previously suggested that RSC is required for efficient *GAL1/GAL10* gene induction only when cells were shifted from a repressive condition (glucose) but not from non-induced conditions (raffinose) to galactose [7]. In Δ*dia2* cells, however, we observed a delay in *GAL1* induction when we added galacotse to cultures grown in raffinose. Rsc3 constitutes one of RSC's DNA binding subunits. Although we observed a reduction in the amount of Rsc3 present in the RSC complex (Fig. 5), it was not absent and recruitment of the core RSC subunits to chromatin was largely independent of Dia2. Therefore, the defect observed in the absence of Dia2 is likely due to the qualitative difference in RSC complex composition rather than its quantitative absence. This may account for the differences between published studies using mutations in essential RSC core components and our observations using Δ*dia2* cells. In addition, we cannot exclude that Dia2 targets chromatin remodellers or transcriptional regulators in addition to the RSC complex.

Dia2 forms part of the SCF^Dia2^ complex but its substrates have proven elusive. Work on replication [10] has identified Mrc1 as a candidate substrate responsible for the role of Dia2 in replication. In the fission yeast *S. pombe*, deletion of the Dia2 homologue, Pof3, demonstrates a variety of functions, which closely resemble those of Dia2 including accumulation of DNA damage and checkpoint activation [27], [28]. Recently, Pof3 has been shown to control histone gene transcription by degradation of a *pombe*-specific histone gene transcription activator Ams2 [29], [30]. While the evolutionary conservation of Ams2 is uncertain, it is intriguing that Dia2 in *S. cerevisiae* is similarly involved in controlling histone gene transcription. The ubiquitylation target of Dia2 responsible for RSC complex assembly is as yet unknown. Our mass spectrometric data is consistent with the possibility that RSC subunits themselves are targeted by Dia2. Confirmation of this possibility, and the question of how ubiquitylation aids RSC complex assembly, will be interesting topics for further investigations.

Our biochemical analysis demonstrated that Dia2 is required specifically for association of a sub-module of the RSC complex, including Htl1, Rtt102 and partially Rsc3, with the core chromatin remodelling complex. Most likely as a consequence, defects in nucleosome remodelling were observed at two model loci investigated. Other reported functions of the RSC complex at these loci remained intact. This opens the possibility that sub-modules within the RSC complex convey specific functions of the complex in the remodelling of nucleosomes during transcriptional activation and repression. Given that SCF^Dia2^ participates in controlling genome stability at several levels, it will be interesting to investigate in how far this is related to its role in controlling RSC assembly, as also the RSC complex participates in mediating DNA repair and chromosome stability in several ways [11], [31]. Further work is required to investigate whether SCF^Dia2^ also controls assembly of other chromatin remodellers.

## Materials and Methods

### Yeast Strains

*S. cerevisiae* strains used are listed in Table S1 in the supplemental data. Standard protocols were used for yeast culture and propagation [32]. The Dia2-HA construct was created using a PCR-based gene targeting method [33].

### Genetic interaction with RSC complex

RSC degron strains were constructed and provided by the Logie lab [11]. Briefly, wild-type or the degron-containing strains were transformed with Δ*suc2* (control strain) or Δ*dia2*. Transformants were split onto plates containing either YPD (2% dextrose), in which the degron remains inactive, and YPG (2% galactose), in which the degron is activated by virtue of induced Ubr1 expression from *pGAL1*. G418 (for selection of knockout transformants) and 0.1 mM copper sulphate (to drive expression of the degron tagged RSC subunit) were also included in all media used. Plates were then incubated for 2 days at 30°C to allow colonies to develop. Colony counts were taken as a measure of viability following each transformation. Relative viability =  No. of colonies on test sample on YPG / No. colonies on YPD in the respective backgrounds. In galactose, where the degron was active, relative viability denoted the effect of growth inhibition in combination with the Δ*dia2* background. The control Δ*suc2* did not result in reduced relative viability demonstrating that degron activation alone could not account for the results observed in the Δ*dia2* background.

### Chromatin Immunoprecipitation

Yeast cells were harvested at O.D._600_ = 1.0 and treated with 1% formaldehyde for 30 minutes at room temperature for crosslinking. Cross-linking was halted by addition of 125 mM glycine for 5 minutes. Lysate preparation and immunoprecipitation of HA-tagged proteins was carried out in accordance with Ren *et al*. [34] using the 12CA5 anti-HA monoclonal antibody. Lysate preparation and immunoprecipitation of TAP-tagged proteins was carried out in accordance with standard protocols [35]. Eluants after crosslink reversal were analysed by qPCR using a Biorad MiniOpticon system. PCR oligo sequences are available upon request.

### RNA Extraction and Analysis

Total RNA was extracted using the ‘RNeasy’ kit (Qiagen) and the corresponding RNase-free DNase (Qiagen). Equal amounts of total RNA were used as template for RT-qPCR to quantify *GAL1, HTA1*, *HTA2, HHF1* or *HHF2* expression, using primers specific to these transcripts. Primers specific to the *ACT1* or *TCM1* transcripts were used for normalization. Expression levels relative to the control were obtained using the following formula y = [2^−C(t)^/2^−control C(t)^]×100.

### Tandem Affinity Purification and Analysis by Tandem Mass Spectrometry

Tandem affinity purification of TAP-tagged proteins, Sfh1 and Htl1, was carried out as described in Puig *et al*. [36]. Eluates were prepared for tandem mass spectrometry using methanol/chloroform precipitation and in-solution trypsin digestion as described previously [37], [38]. In brief, proteins were desalted and concentrated by methanol/chloroform precipitation, resuspended in 6 M urea/100 mM Tris pH 7.8, reduced using 20 mM dithiothreitol, alkylated using 20 mM iodoacetamide, reduced again with excess dithiothreitol to neutralize unreacted iodoacetamide, diluted five-fold with 100 mM Tris pH 7.8, digested with trypsin (Promega) and purified using Sep-Pak C18 columns (Waters). Samples were concentrated in vacuo, resuspended in 2% acetonitrile/0.1% formic acid and stored at −20°C until analysis. Analysis by tandem mass spectrometry was conducted using a nano-Acquity-QToF tandem mass spectrometer (nano-UPLC-MS, Waters) essentially as reported previously [39]. MS/MS spectra were searched against the Swissprot database using Mascot. Semiquantitative information about differential abundance of protein complex components was obtained by evaluating their exponentially modified protein abundance index (EmPAI) as described previously [40].

### Immunoblotting Assays

Proteins were separated by SDS-PAGE and fixed overnight in 50% methanol for silver staining. For western blotting, SDS-PAGE gels were transferred to PVDF membranes according to a standard protocol. Antibodies used: Anti-HA (12CA5) 1∶1000, anti-calmodulin binding protein (Upstate, Clone C16T) = 1∶5000 and anti-ubiquitin (monoclonal FK2; Abcam, UK)  = 1∶1000.

### Nucleosome Positioning Assay

Nucleosome positioning was assessed using an assay based on Bryant *et al.* [25]. Micrococcal nuclease-digested DNA was prepared according to Liu [41]. Briefly, cells were harvested at O.D._600_ = 1.0 and treated with 1% formaldehyde for 30 minutes at room temperature, followed by 125 mM glycine for 5 minutes. Cells were then treated with zymolyase (Seikagaku Biobusiness Corporation) in Buffer Z (1 M sorbitol, 50 mM Tris-Cl [pH 7.4]), 28 µl of β-ME (14.3 M, final concentration 10 mM) to digest cell walls and gently lysed using a hypotonic buffer (0.5 mM spermidine, 1 mM β-ME, 0.075% NP-40, 50 mM NaCl, 10 mM Tris [pH 7.4], 5 mM MgCl_2_, 1 mM CaCl_2_). Chromatin was digested using micrococcal nuclease (USB Corporation) to achieve mononucleosomal-sized fragments. DNA was then extracted by two rounds of phenol/chloroform, followed by ethanol precipitation.

Micrococcal nuclease-digested DNA, and undigested genomic DNA were analysed by qPCR, using a panel of primers corresponding to the specified promoters and 5′ parts of the open reading frames. Data was normalised against undigested controls (y = [2^−MNase^ ^C(t)^/2^−undigested C(t)^]×100), then internally against a non-nucleosomal control region [25], [42], [43]. Oligos used for nucleosome mapping are available upon request.

## Supporting Information

Figure S1 **Dia2 shows specific genetic interaction with some but not all RSC subunits.** Dataset for all *rsc^td^* subunits tested in combination with Δ*dia2* or the control KO cassette (Δ*suc2)* that has been demonstrated by the Logie group to not interact genetically with the RSC complex. Relative viability is shown during degron-inducing (galactose) conditions corrected to viability in degron non-inducing (dextrose) conditinos. The *rsc3^td^* mutation conferred reduced viability relative to wild-type in both the Δ*dia2* and the control strain under inducing conditions. As such, it was impossible to deduce whether the potential interaction observed was genuine.
(EPS)

Figure S2 **Transcription of histone genes in the Δ** ***dia2*** **mutant.** Total RNA was extracted from yeast cells grown in YPD either in the absence (asyn) or after addition of 200 mM of hydroxyurea (HU) for 40 minutes to induce repression. RNA was reverse transcribed and analysed by quantitative PCR. Samples were normalised against expression of the *ACT1* gene. Gene expression at the *HTA1*, *HTA2* (representative of transcription of the histone H2A and H2B genes), *HHF1* and *HHF2* (representative of transcription of the histone H3 and H4 genes) loci were shown.
(EPS)

Figure S3 **Dia2 is required for correct nucleosome positioning at the** ***HTA1/HTB1*** **promoter.** S3A) Diagramatic representation of predicted nucleosome and primer locations on the *HTA1/HTB1* promoter. S3B) Nucleosome mapping by micrococcal nuclease protection assays in wild-type or Δ*dia2* cells grown in YPD (‘asyn’: triangles) or in the presence of 200 mM hydroxyurea (HU: circles). Error bars represent standard error of the mean from three biological replicates. Statistical significance is <0.0001 when comparing entire data sets- determined by 2-way ANOVA. During hydroxyurea-mediated repression, there is an expected increase in nucleosome deposition (top panel). This is particularly evident between primer positions 12–18 in the wild-type strain. In the Δ*dia2* strain however, nucleosome boundaries were generally less sharply defined (broader peaks, bottom panel). There is also a lack of protection to micrococcal nuclease digestion during repression suggestive of more open chromatin structure and less nucleosome deposition.
(EPS)

Figure S4 **Htl1 is expressed in the Δ** ***dia2*** **mutant though it no longer associates with the RSC complex.** Tandem affinity purification was performed using TAP-tagged Htl1 in the wild-type (WT) or the Δ*dia2* backgrounds. Left panel: Anti-calmodulin-binding protein (Anti-CBP) blot to indicate that immunoprecipitation was equally efficient in both samples and that Htl1 is adequately expressed in the absence of Dia2. Right panel: Western blotting using an anti-ubiquitin antibody (Anti-Ubq). Multiple ubiquitylated proteins were observed in the wild-type sample and some of these are absent in Δ*dia2* cells, indicated by *s. This reflected that the RSC complex was not efficiently pulled down in Δ*dia2* cells.
(EPS)

Table S1 **List of yeast strains employed in this study.**
(DOC)
