# Supplementary material for: Dia2 Controls Transcription by Mediating Assembly of the RSC Complex
Source: PLoS One. 2011 Jun 20;6(6):e21172. doi: 10.1371/journal.pone.0021172 (PMC3118812; doi:10.1371/journal.pone.0021172)
Supplement: Table S1 — List of yeast strains employed in this study. (DOC) [file pone.0021172.s005.doc]

| **Strain** | **Genotype** | **Source** |
| --- | --- | --- |
| WT (BY4741) | *MATa; his3; leu2; met15; ura3* | Euroscarf |
| *Δdia2* | *MATa; his3; leu2; met15; ura3; YOR080w::kanMX4* | Euroscarf |
| Dia2-HA | *MATa; his3; leu2; met15; ura3; Dia2-HA::TRP1* | This study |
| Sfh1-TAP | *MATa; ade2; arg4; leu2-3,112; trp1-289; ura3-52; Sfh1-TAP::URA3* | Opensystems |
| Htl1-TAP | *MATa; ade2; arg4; leu2-3,112; trp1-289; ura3-52; Ht1-TAP::URA3* | Opensystems |
| Rsc8-TAP | *MATa; ade2; arg4; leu2-3,112; trp1-289; ura3-52; Rsc8-TAP::URA3* | Opensystems |
| Sfh1-TAP *Δdia2* | *MATa; ade2; arg4; leu2-3,112; trp1-289; ura3-52; Sfh1-TAP::URA3; YOR080w::kanMX4* | This study |
| Htl1-TAP *Δdia2* | *MATa; ade2; arg4; leu2-3,112; trp1-289; ura3-52; Ht1-TAP::URA3; YOR080w::kanMX4* | This study |
| Rsc8-TAP *Δdia2* | *MATa; ade2; arg4; leu2-3,112; trp1-289; ura3-52; Rsc8-TAP::URA3; YOR080w::kanMX4* | This study |
| YN96 (WT) | *MATa; trp1-1; ura3-1; his3-11,15; leu2-3, 112PGAL1-10-myc::UBR1-HIS3* | C. Logie |
| YN119  (Sth1 degron) | *MATa; trp1-1; ura3-1; his3-11,15; leu2-3, 112; PGAL1-10-myc::UBR1-HIS3; Δlys2::rKWD50N;*  *PCUP1-degron::sth1::URA3* | C. Logie |
| YN147  (Rsc3 degron) | *MATa; trp1-1; ura3-1; his3-11,15; leu2-3, 112; PGAL1-10-myc::UBR1-HIS3; Δlys2::rKWD50N;*  *PCUP1-degron::rsc3::URA3* | C. Logie |
| YN159  (Rsc8 degron) | *MATa; trp1-1; ura3-1; his3-11,15; leu2-3, 112;*  *PGAL1-10-myc::UBR1-HIS3; Δlys2::rKWD50N; PCUP1-degron::rsc8::URA3* | C. Logie |
| YN218  (Rsc4 degron) | *MATa; trp1-1; ura3-1; his3-11,15; leu2-3, 112; PGAL1-10-myc::UBR1-HIS3; Δlys2::rKWD50N; PCUP1-degron::rsc4::URA3* | C. Logie |
| YN220  (Sfh1 degron) | *MATa; trp1-1; ura3-1; his3-11,15; leu2-3, 112; PGAL1-10-myc::UBR1-HIS3; Δlys2::rKWD50N;PCUP1-degron::sfh1::TRP1* | C. Logie |
| YN315  (Rsc58 degron) | *MATa; trp1-1; ura3-1; his3-11,15; leu2-3, 112; PGAL1-10-myc::UBR1-HIS3; Δlys2::rKWD50N; PCUP1-degron::rsc58::TRP1* | C. Logie |
| YN319  (Rsc6 degron) | *MATa; trp1-1; ura3-1; his3-11,15; leu2-3, 112PGAL1-10-myc::UBR1-HIS3; Δlys2::rKWD50N; PCUP1-degron::rsc6::LEU2* | C. Logie |
| YN321  (Rsc9 degron) | *MATa; trp1-1; ura3-1; his3-11,15; leu2-3, 112; PGAL1-10-myc::UBR1-HIS3; Δlys2::rKWD50N;*  *PCUP1-degron::rsc9::TRP1* | C. Logie |

Table S1: Yeast strains used in this study
